# Mothers' Knowledge, Attitude, and Behavior Concerning Their Kindergarten Children's Oral Health: A Cross‐Sectional Study

**DOI:** 10.1002/cre2.70113

**Published:** 2025-03-11

**Authors:** Dhuha K. Qahtan, Osama M. Felemban, Rana A. Alamoudi, Nada O. Bamashmous, Eman A. El Ashiry, Najlaa M. Alamoudi

**Affiliations:** ^1^ Department of Pediatric Dentistry, Faculty of Dentistry King Abdulaziz University Jeddah Saudi Arabia; ^2^ Private Practitioner of Pediatric Dentistry, Medical Fakeeh Jeddah Saudi Arabia; ^3^ Department of Pedodontics and Oral Health, Faculty of Dental Medicine for Girls Al Azhar University Cairo Egypt

**Keywords:** attitude, dental caries, health knowledge, oral health, practice, preschool children

## Abstract

**Objectives:**

To evaluate the level of oral health‐related knowledge, attitudes, and behavior among a group of mothers with kindergarten (KG) children aged 3–5 years toward their own and their children's oral health and assess its influence on their children's oral health status.

**Material and Methods:**

This was a cross‐sectional study conducted in Jeddah, Saudi Arabia. The sample was selected randomly from public and private KGs in Jeddah. Self‐administrated questionnaires were distributed to the mothers of KG schoolchildren aged 3–5 years, which contained translated and validated Mothers' Behavior Questionnaire about their own oral health behaviors, Mothers' Attitude Questionnaire about their children's oral health, and Mothers' Knowledge Questionnaire about their children's oral health. The oral health of the KG school children was examined to determine the decayed, missed, and filled index (dmft).

**Results:**

A total of 461 child–mother pairs completed the study. The mean values of dmft were 5.41 ± 4.81. The children's oral health (dental caries) and the mothers' oral health‐related knowledge, attitudes, and behavior were significantly associated with KG type (public vs. private), mothers' age, mothers' education, and family income. A multiple linear regression model indicated that younger mothers (< 30–40 years), highly educated mothers, high family income, and mothers with higher knowledge scores were significantly associated with lower dmft scores.

**Conclusions:**

Mothers whose children attended private KGs exhibited better oral health‐related attitudes, habits, and knowledge. School type, mother's age, mother's education level, and monthly income were factors that strongly impacted the behaviors, attitudes, and knowledge of the mothers. Dental caries was lower among children whose mothers were young, well‐educated, from high family income families, and had higher knowledge related to oral health. Implementing targeted educational programs for mothers, particularly those with lower educational attainment and from low‐income backgrounds, is essential for enhancing the oral health of children in kindergarten age.

## 1 Introduction

Oral health is essential for an individual's overall health and quality of life. Poor oral health can affect socio‐behavior activity, impair nutritional status, and affect the growth of children. One of the most severe oral health issues worldwide is dental caries. Increased incidence of dental caries is associated with different factors such as parents' education, socioeconomic status, and their knowledge and attitudes toward their children's oral health (Kuppuswamy et al. 2014).

Insufficient knowledge and inadequate practice regarding oral health and hygiene of their children can result in a higher risk of dental caries in young children (Alhabdan et al. 2018; Bedaiwi et al. 2017; Mehta et al. 2019). Parents tend to be their children's role models, especially in oral hygiene practice (Nepaul and Mahomed 2020). A strong association between the oral health of the mother and their children has been reported earlier (Lee et al. 2019). Similarly, a direct connection between children's dental health conditions and their mothers' educational levels has been found (Kuppuswamy et al. 2014; Quadri et al. 2015). Mothers with low educational levels (less than secondary school education) had poor knowledge regarding children's oral health (Moses and Arunachalam 2018), while mothers with a high level of knowledge and attitudes toward oral health had children with good dental health (Bozorgmehr et al. 2013). However, some contradicting results showed that parents' attitudes toward their children's oral health were poor, but the children were found to have good oral health (Nepaul and Mahomed 2020).

There is a relationship between the dental health status of children and their educational performance. Children with poor oral health are associated with low grades at school because severe dental pain, dental abscesses, and infections result in children missing school (Anandakrishna and Chandra 2012). This is reflected in poor academic performance and lower school levels of children with poor oral health compared to those with good oral health. Poor oral health can also have financial implications and affect the child's confidence (Anandakrishna and Chandra 2012; Mehta et al. 2019; Renzaho and de Silva‐Sanigorski 2014). It can also adversely affect these children's quality of life and their families (Abreu et al. 2021).

Several authors have studied mothers' knowledge and attitudes toward their children's oral health (Gerreth et al. 2020; Mahmoud et al. 2017; Nepaul and Mahomed 2020; Prabhu et al. 2013). The significant role of mothers' knowledge has been found in preventing dental caries and poor oral hygiene of their children in various provinces of Saudi Arabia (Al‐Zahrani et al. 2014; Alhabdan et al. 2018; Alshehri 2016). However, there is limited research investigating mothers' knowledge and attitude concerning their children's oral health in Jeddah, Saudi Arabia. One study conducted in Saudi Arabia evaluates the relationship between parent behavior and attitudes toward their children's oral health and oral hygiene status (Alhabdan et al. 2018). Mothers play a vital role in preventing dental caries by influencing their children's oral health behaviors through their knowledge, attitudes, and practices. Understanding these maternal factors is crucial for assessing their impact on children's oral health outcomes and developing effective preventive strategies. Therefore, the present study aimed to evaluate the level of oral health‐related knowledge, attitudes, and behavior among mothers who have children studying in Kindergarten (KG), aged 3–5 years, toward their own oral health and their children's oral health and assess the influence of the knowledge, attitude, and behavior on the oral health status of the children.

## 2 Methodology

This cross‐sectional study was conducted in kindergarten (KG) schools in Jeddah, Saudi Arabia. The research protocol was approved by the Research Ethics Committee at the Faculty of Dentistry, King Abdulaziz University, Saudi Arabia with a proposal number (008‐16). Local School Health and Education Directorate Authority, Ministry of Education was approached for the lists of the KG in the Jeddah district.

The target population of the study was mothers and their children aged 3–5 years who were medically healthy, Arabic speaking (because the questionnaire was in Arabic), and attending either private or public KGs in the city of Jeddah, Saudi Arabia. It was anticipated that 406 individuals from public and private schools would be needed to get accurate findings with a confidence level of 90% ± 5%, assuming a hypothesized outcome frequency of 25%. A multistage stratified random sampling technique was used to identify the participants from KG schools. For the sake of ensuring a balanced sampling of geographical location and socioeconomic status, the KGs were stratified by the district into four strata (west, east, north, and south) and further into KG management types (public/private), resulting in eight strata. Kindergarten (KG) schools were listed in each of the eight strata. One KG school was selected at random from each list using a random number generator, resulting in a total of eight KG schools. Following the random selection of the participating schools, the principals of each participating school were contacted to discuss the primary idea of the research and obtain permission to visit the school and carry out the study. Three classes, one from each grade (KG1, KG2, and KG3), were randomly selected from each KG school using the bowl technique. Every class ought to have a minimum of 20 students. If any class had fewer than 20 children, another class was chosen at random. The children in the classes and their mothers were invited to participate in the study. The class teacher facilitated the distribution of the questionnaires to the selected children, which included informed consent and permission to examine their teeth. This school visit was considered the first visit. The children took the questionnaires home for the mothers to sign and fill out. The utilization of self‐administered questionnaires facilitated the inclusion of a substantial sample size. All of the data obtained from the participants was anonymous, and only the principal investigator had access to the data.

The questionnaire consisted of four sections:

1‐ Demographic data include questions regarding potential confounders such as age, gender, educational background, and familial income.
2‐ The Mothers' Behavior Questionnaire (MBQ) evaluated the mothers' behaviors related to their dental health, utilizing the Hiroshima University – Dental Behavioral Inventory (HU‐DBI) questionnaire (Kawamura 1988). The HU‐DBI questionnaire evaluated the opinions and behaviors of the participating women toward their oral health. It consisted of 20 items presented in a dichotomous format, where participants could respond with either “agree” or “disagree.” An adjustment was made to item number 5, where the phrase (I use a child‐sized toothbrush) was changed to (I use a recommended‐sized toothbrush). This modification was implemented to enhance accuracy and accommodate specifically mothers.
3‐ The Mothers' Attitude Questionnaire (MAQ) was utilized to evaluate the oral health‐related attitude of mothers toward the oral health of their children. This questionnaire was based on a modified version of a prior questionnaire (Lenčova 2013). This study specifically focused on the 13 items that examine the potential impact of mothers' attitudes toward oral health on their children's dental health.
4‐ The Mothers' Knowledge Questionnaire (MKQ) evaluated the oral health‐related knowledge of mothers on their children's oral health. It utilized a modified version of a prior questionnaire (Prabhu et al. 2013), which assessed maternal knowledge regarding children's oral health, dental care, and dietary habits. Modifications were made to this component, resulting in the usage of only 12 questions instead of the original 14. Specifically, two questions were removed, which asked whether the child under the age of five had visited a dentist and, if so, what procedures were performed. These two questions pertain to behavior rather than knowledge. Within this section of the questionnaire, the mothers who took part selected the best answer from multiple choices.

The questionnaire was translated into Arabic by two linguists using forward and backward translation methods. Subsequently, the two linguists and a dental public health specialist compared the original English questionnaire with the translated version and made necessary modifications. To evaluate test–retest reliability, 20 mothers who were not part of the study were asked to complete the final Arabic version of the questionnaire twice, separated by a period of 2 weeks. The results were evaluated using Pearson's correlation coefficient (r) as a measure of reliability, yielding a value of 0.9, which was considered to be excellent. The internal consistency of the final Arabic‐translated version of the three sections of the questionnaire was assessed using Cronbach's Alpha, yielding a value of 0.86. A Cronbach's alpha of 0.70 or higher indicates sufficient internal consistency. The content validity index (CVI) was computed using an established approach (Polit et al. 2007; Lynn 1986) to determine if the items in the questionnaires accurately represented the complete theoretical construct that the questionnaire aimed to evaluate. The survey was disseminated to a group of specialists in the field of pediatric dentistry. The panel of experts was requested to evaluate each item in the questionnaire using a four‐point Likert scale, considering factors such as relevance, clarity, simplicity, and ambiguity. The content validity was determined to be 0.84. The assessment of construct validity involved the correlation of individual questions with the total score of each section of the questionnaire using Spearman's rho. The construct validity values were 0.83, 0.72, and 0.76.

The second visit took place 1 week later to collect the questionnaire and do a dental examination on the children. The dmft index was used to assess the severity of caries in the primary teeth. The participants' oral and gingival health was assessed using the Simplified Oral Hygiene Index (OHI‐S) (Greene and Vermillion 1964). The dental examination employed the “Tell‐Show and Do” technique. Thereafter, a brief report was issued to mothers detailing their child's oral health condition and treatment requirements. A total of four examiners who were general dentists underwent calibration and training. A total of 20 pediatric dental patients were selected from pediatric dentistry clinics at the KAU Dental Hospital for assessing dental caries (dmft) according to WHO criteria. The recorded data were compared among examiners to assess inter‐rater reliability using the Intra‐Class Correlation (ICC) coefficient, which yielded a value of 0.96. One week later, the same group of patients underwent a second examination. This was done to assess the intra‐rater reliability of each examiner, which resulted in values ranging from 0.93 to 0.95.

A third visit was done in the same week where the authors met the participating mother–child pairs to present oral health educational lectures and distribute oral health instruction pamphlets.

The data was analyzed using the Statistical Package for Social Science (SPSS), specifically IBM Statistics for Windows, Version 23.0, developed by IBM Corp in Armonk, NY. The data analysis did not include the small number of questionnaire responses that had missing data. The chi‐square test was implemented to compare the questionnaire responses of participants from public and private schools. Total scores of correct answers for each questionnaire were calculated where higher scores indicated superior knowledge, attitude, and behavior. The range for the questionnaire scores was 0–20 for the MBQ, 0–13 for the MAQ, and 0–12 for the MKQ. The associations between demographic characteristics and the independent and dependent variables were assessed using an independent sample *t*‐test and one‐way ANOVA. To determine the impact of various independent variables (KG type) on the dependent variable (total dmft), while accounting for potential confounding factors (child gender, mother age, mother education level, monthly income, questionnaire scores, and OHI‐S), a multiple linear regression analysis was conducted.

## 3 Results

Out of the 480 distributed questionnaires, 461 child–mother pairs returned the questionnaire, and their children were dentally examined yielding a response rate of 96%. The demographic attributes of the children and mothers are presented in Table 1.

**Table 1:** Demographic characteristics of the children and their mothers in the study sample.

Variable	Category	Frequency (%)
**School type**	**Public**	256 (55.5)
	**Private**	205 (44.5)
**Child age**	**3–4 years**	232 (50.3)
	**5 years**	229 (49.7)
**Child gender**	**Male**	222 (48.2)
	**Female**	239 (51.8)
**Mother's age**	**30 or less**	130 (28.2)
	**31–40**	233 (50.5)
	**41 or more**	98 (21.3)
**Mother's education**	**High school or less**	206 (44.7)
	**Diploma/University or higher**	255 (55.3)
**Monthly Income**	**Low**	112 (24.3)
	**Medium**	234 (50.8)
	**High**	115 (24.9)

The mean dmft and OHI‐S debris Index were 5.41 ± 4.81 and 0.94 ± 0.97, respectively. Table 2 compares the responses of the mothers to the Mothers' Behavior Questionnaire (MBQ) between public and private KGs. Of the 20 questions, there was a statistically significant difference in 18 questions between mothers of children in public compared to mothers of children in private KGs (*p* < 0.001) where more positive oral health behavior was reported by mothers listed in private KGs.

**Table 2:** Comparison of the responses of the mothers to the Mothers' Behavior Questionnaire (MBQ) between public and private schools.

Items		The correct answer	Total (*n* = 461)	Public (*n* = 256)	Private (*n* = 205)	*p* value
**1**	**I don't worry much about visiting the dentist**	**Agree**	230 (49.9)	111 (43.4)	119 (58.0)	0.002
**2**	**My gums tend to bleed when I brush my teeth**	**Disagree**	334 (72.5)	164 (64.1)	170 (82.9)	< 0.001
**3**	**I worry about the color of my teeth**	**Agree**	269 (58.4)	176 (68.8)	93 (45.4)	< 0.001
**4**	**I have noticed some white sticky deposits on my teeth**	**Agree**	40 (8.7)	31 (12.1)	9 (4.4)	0.003
**5**	**I use a recommended‐sized toothbrush**	**Agree**	445 (96.5)	242 (94.5)	203 (99.0)	0.009
**6**	**I think that I cannot help having false teeth when I am old**	**Disagree**	34 (7.4)	29 (11.3)	5 (2.4)	< 0.001
**7**	**I am bothered by the color of my gums**	**Disagree**	386 (83.7)	201 (78.5)	185 (90.2)	0.001
**8**	**I think my teeth are getting worse despite my daily brushing**	**Disagree**	291 (63.1)	141 (55.1)	150 (73.2)	< 0.001
**9**	**I brush each of my teeth carefully**	**Agree**	362 (78.5)	191 (74.6)	171 (83.4)	0.022
**10**	**I have never been professionally taught how to brush**	**Disagree**	179 (38.8)	92 (35.9)	87 (42.4)	0.155
**11**	**I think I can clean my teeth without using toothpaste**	**Agree**	151 (32.8)	55 (21.5)	96 (46.8)	< 0.001
**12**	**I often check my teeth in a mirror after brushing**	**Agree**	337 (73.1)	174 (68.0)	163 (79.5)	0.005
**13**	**I worry about having bad breath**	**Agree**	86 (18.7)	58 (22.7)	28 (13.7)	0.014
**14**	**It is impossible to prevent gum disease without toothbrushing alone**	**Disagree**	153 (33.2)	70 (27.3)	83 (40.5)	0.003
**15**	**I put off going to the dentist until I have a toothache**	**Disagree**	75 (16.3)	25 (9.8)	50 (24.4)	< 0.001
**16**	**I have used a dye to see how clean my teeth are**	**Agree**	9 (2.0)	7 (2.7)	1 (1.0)	0.175
**17**	**I use a toothbrush that has hard bristles**	**Disagree**	374 (81.1)	184 (71.9)	190 (92.7)	< 0.001
**18**	**I don't feel I've brushed well unless I brush with strong strokes**	**Disagree**	279 (60.5)	121 (47.3)	158 (77.1)	< 0.001
**19**	**I feel I sometimes take too much time to brush my teeth**	**Agree**	251 (54.4)	106 (41.4)	145 (70.7)	< 0.001
**20**	**I have had my dentist tell me that I brush very well**	**Agree**	164 (35.6)	69 (27.0)	95 (46.3)	< 0.001

*Note:* Chi‐square test.

Table 3 features the differences in responses to the Mother's Attitude Questionnaire (MAQ) between public and private KG mothers. Most of the MAQ questionnaire showed a statistically significant difference between public and private KGs and mostly in favor of the mothers with children in private KGs.

**Table 3:** Comparison of the responses to the Mother's Attitude Questionnaire (MAQ) between public and private schools.

Items		The correct answer	Total (*n* = 461)	Public (*n* = 256)	Private (*n* = 205)	*p* value
**1**	**We feel it is important that we check our child's teeth for decay**	**Agree**	452 (98.0)	254 (99.2)	198 (96.6)	0.042
**2**	**I don't know how to brush my child's teeth properly**	**Disagree**	292 (63.3)	144 (56.3)	148 (72.2)	< 0.001
**3**	**We feel it is important to check if our child has brushed his/her teeth**	**Agree**	442 (95.9)	247 (96.5)	195 (95.1)	0.465
**4**	**we don't have time to help brush our child's teeth daily**	**Disagree**	298 (64.6)	151 (59.0)	147 (71.7)	0.005
**5**	**It is the responsibility of the dentist to prevent our child from getting tooth decay**	**Disagree**	174 (37.7)	110 (43.0)	64 (31.2)	0.010
**6**	**If our child gets tooth decay, it is by chance**	**Disagree**	304 (65.9)	156 (60.9)	148 (72.2)	0.011
**7**	**It would not make any difference to our child getting tooth decay if we helped him/her brush every day**	**Disagree**	135 (29.3)	63 (24.6)	72 (35.1)	0.014
**8**	**It is worthwhile to give our child sweets/biscuits to behave well**	**Disagree**	207 (44.9)	77 (30.1)	130 (63.4)	< 0.001
**9**	**Tooth decay is a serious problem in baby teeth**	**Agree**	352 (76.4)	171 (66.8)	181 (88.3)	< 0.001
**10**	**As parents, it is our responsibility to prevent our child from getting tooth decay**	**Agree**	449 (97.4)	252 (98.4)	198 (96.1)	0.117
**11**	**We can prevent tooth decay in our child by reducing sugary foods and drinks between meals**	**Agree**	445 (96.5)	243 (94.9)	202 (98.5)	0.035
**12**	**If we brush our child's teeth daily, we can prevent our child from getting tooth decay in the future**	**Agree**	356 (77.2)	185 (72.3)	171 (83.4)	0.005
**13**	**If our child uses a fluoride toothpaste, it will prevent tooth decay**	**Agree**	395 (85.7)	203 (79.3)	192 (93.7)	< 0.001

*Note:* Chi‐square test.

Table 4 compares the Mothers' Knowledge Questionnaire (MKQ) about oral health and proper oral hygiene between mothers of public and private KGs. In general, mothers of children in private KGs demonstrated significantly superior knowledge about oral health and best oral hygiene practices compared to mothers of children in public KGs.

**Table 4:** Comparison of the responses to the Mothers' Knowledge Questionnaire (MKQ) between public and private schools.

Items		The correct answer	Total (*n* = 461)	Public (*n* = 256)	Private (*n* = 205)	*p* value
**1**	**How often should you brush your child's teeth**	**Twice daily**	232 (50.3)	110 (43.0)	122 (59.5)	< 0.001
**2**	**Size of brush best for your child**	**Small**	397 (86.1)	213 (83.2)	184 (89.8)	0.043
**3**	**Quantity of paste to be used**	**Pea size**	160 (34.7)	110 (43.0)	50 (24.4)	< 0.001
**4**	**Your position to brush your child's teeth**	**Behind the child**	86 (18.7)	26 (10.2)	60 (29.3)	< 0.001
**5**	**Does your child's toothpaste have fluoride**	**Yes**	335 (72.7)	166 (64.8)	169 (82.4)	< 0.001
**6**	**Fluoride content of child paste**	**1000 or 1450 ppm**	36 (7.8)	13 (5.1)	23 (11.2)	0.015
**7**	**Four of the following cause tooth decay.**		396 (85.9)	200 (78.1)	196 (95.6)	< 0.001
	**Chocolate**	**Correct**				
	**Cheese**					
	**Biscuits**	**Correct**				
	**Sweets**	**Correct**				
	**Soft drink**	**Correct**				
**8**	**Best time to give sugary snacks**	**Mealtime**	87 (18.9)	31 (12.1)	56 (27.3)	< 0.001
**9**	**Has the child used a sweetened baby bottle or honey‐dipped pacifier**	**No**	384 (83.3)	196 (76.6)	188 (91.7)	< 0.001
**10**	**Importance of decay in baby teeth**	**Very important**	423 (91.8)	222 (86.7)	201 (98.0)	< 0.001
**11**	**Child's first dental visit**	**On getting first baby tooth**	36 (7.8)	20 (7.8)	16 (7.8)	0.998
**12**	**If baby teeth decayed, what treatment would you prefer**	**Fill it**	355 (77.0)	171 (66.8)	184 (89.8)	< 0.001

*Note:* Chi‐square test.

Associations between demographic variables and MBQ score, MKQ score, MAQ score, and dmft are illustrated in Table 5. Mothers whose children were in private KG had higher scores on the three questionnaires and lower mean dmft. The questionnaire scores decreased as the mothers' age increased; however, the mean dmft increased as the mothers' age increased. Mothers with higher education levels had higher questionnaire scores, but their children had lower mean dmft. The questionnaire scores increased as the income increased, but the mean dmft decreased.

**Table 5:** Associations between demographic variables and the MBQ score, MKQ score, MAQ score, and dmft.

Variable	Category	MBQ	MKQ	MBQ	dmft
**School type**	Public	10.48 ± 3.00	5.77 ± 1.79	8.81 ± 1.83	6.63 ± 5.06
	Private	13.47 ± 3.05	7.07 ± 1.64	9.97 ± 1.57	3.89 ± 3.97
	*p* value^a^	< 0.001	< 0.001	< 0.001	< 0.001
**Child age**	3–4 years	11.72 ± 3.34	6.27 ± 1.97	9.25 ± 1.82	5.12 ± 4.83
	5 years	11.90 ± 3.40	6.43 ± 1.69	9.42 ± 1.81	5.72 ± 4.78
	*p* value^a^	0.576	0.361	0.317	0.181
**Child gender**	Male	11.85 ± 3.19	6.45 ± 1.69	9.36 ± 1.74	5.03 ± 4.39
	Female	11.77 ± 3.52	6.26 ± 1.96	9.29 ± 1.88	5.77 ± 5.14
	*p* value^a^	0.806	0.254	0.652	0.099
**Mother's age**	30 or less	13.25 ± 3.11 a	6.94 ± 1.87 a	9.98 ± 1.55 a	3.73 ± 4.41 a
	31–40	11.46 ± 3.15 b	6.24 ± 1.74 b	9.11 ± 1.83 b	5.41 ± 4.67 b
	41 or more	10.71 ± 3.56 b	5.83 ± 1.80 b	8.97 ± 1.86 b	7.65 ± 4.76 c
	*p* value^b^	< 0.001	< 0.001	< 0.001	< 0.001
**Mother's education**	High school or less	9.99 ± 2.98	5.64 ± 1.83	8.60 ± 1.77	7.72 ± 4.70
	Diploma/University or higher	13.28 ± 2.92	6.93 ± 1.63	9.92 ± 1.63	3.55 ± 4.03
	*p* value^a^	< 0.001	< 0.001	< 0.001	< 0.001
**Monthly Income**	Low	8.81 ± 2.59 a	5.07 ± 1.85 a	8.03 ± 1.79 a	9.75 ± 4.24 a
	Medium	11.9 ± 2.56 b	6.41 ± 1.52 b	9.46 ± 1.56 b	4.49 ± 4.05 b
	High	14.4 ± 3.17 c	7.48 ± 1.62 c	10.3 ± 1.56 c	3.06 ± 4.03 c
	*p* value^b^	< 0.001	< 0.001	< 0.001	< 0.001
**Total**		11.81 ± 3.37	6.35 ± 1.84	9.33 ± 1.81	5.41 ± 4.81

*Note:* Means sharing the same alphabetical letter are not statistically different from each other at *p* < 0.05 using post hoc comparisons.

Means that have different alphabetical letters are statistically different from each other at *p* < 0.05 using post hoc comparisons.

Abbreviations: MAQ, Mothers' Attitude Questionnaire; MBQ, Mothers' Behavior Questionnaire; MKQ, Mothers’ Knowledge Questionnaire.

^a^ Independent sample *t*‐test.

^b^ Analysis of variance test (ANOVA).

To model the influences of KG type, mother's age, mother's education, monthly income, the questionnaires scores, and OHI‐S index score on the dmft index, multiple linear regression (adjusted) was used (Table 6). Fifty‐five percent of the variability was explained by the model (*R* ^2^ = 0.551). Children of younger mothers had lower dmft scores compared to children of mothers 41 years of age or older. The dmft scores of children belonging to mothers of low income were higher by 2.53 on average (95 CI 1.12–3.95) compared to children from high‐income families (*p* < 0.001). As the Mother's Knowledge Questionnaire (MKQ) score increased by one, the dmft of the children decreased by −0.34 (95% CI −0.56 to −0.12), and this association was statistically significant (*p* = 0.002). Also, as the OHI‐S index increased by 1, the dmft index significantly increased by 2.00 on average (95% CI 1.62–2.39, *p* < 0.001).

**Table 6:** Multiple linear regression (adjusted) to model the effects of questionnaire scores on caries (dmft) controlling for demographic variables (*R* ^2^ = 0.551).

Independent variables		β ± SE	95% CI		*p* value
			Lower bound	Upper bound	
**School type**	**Public**	0.10 ± 0.45	−0.79	0.99	0.822
	**Private**	Reference			
**Mother's age**	**30 or less**	−1.99 ± 0.47	−2.92	−1.08	< 0.001
	**31–40**	−1.63 ± 0.40	−2.31	−0.73	< 0.001
	**41 or more**	Reference			
**Mother's education**	**High school or less**	0.51 ± 0.39	−0.26	1.29	0.193
	**Diploma/University or higher**	Reference			
**Monthly Income**	**Low**	2.53 ± 0.72	1.12	3.95	< 0.001
	**Medium**	0.19 ± 0.48	−0.76	1.13	0.695
	**High**	Reference			
**MBQ**		−0.07 ± 0.07	−0.22	0.08	0.343
**MKQ**		−0.34 ± 0.11	−0.56	−0.12	0.002
**MAQ**		−0.19 ± 0.12	−0.42	0.04	0.105
**OHI‐S index**		2.00 ± 0.20	1.62	2.39	< 0.001

Abbreviations: β beta estimate; CI, confidence interval; MAQ, Mothers' Attitude Questionnaire; MBQ, Mothers' Behavior Questionnaire; MKQ, Mothers' Knowledge Questionnaire; SE, standard error.

## 4 Discussion

This study highlights the significant impact of maternal knowledge, attitudes, and behaviors on the oral health of kindergarten children. Additionally, some factors play a crucial role in shaping children's oral health, alongside oral health‐related knowledge, attitudes, and behaviors.

The oral health of children can be influenced by the knowledge, attitudes, and behaviors of their parents. Most children under 5 years of age spend their time with their parents or caregivers. The “primary socialization” stage of these initial years develops the earliest routines and habits of children. The role that parents play in ensuring their child's dental health during the first 3 years of preschool is crucial (Samuel et al. 2020). The results of the current study concentrated on mothers' knowledge, attitudes, and practices regarding their own oral health as well as the dental health of their preschool‐aged children in Jeddah, Saudi Arabia. Previous research reported on comparable mother cohorts in Qatar (Alkhtib and Morawala 2018), Iran (Azimi et al. 2018), Indonesia (Abdat and Ramayana 2020), and the western region of Saudi Arabia (Nassar et al. 2022). In the current study, we looked at the mother's age, education level, monthly income, and KG type as variables that affected their knowledge and awareness of dental health and their effects on oral health. Previous research reported these variables (Alhabdan et al. 2018). The findings of our investigation found that the oral health of children was affected negatively by older mothers, lower educational levels, and monthly income.

Dental caries is still a plague on humanity today, despite extensive preventative methods. Globally, this disease is associated with physical, mental, financial, and social burdens, with underdeveloped and developing nations being most severely impacted (Sharna et al. 2019). The dmft is a crucial measure of oral health status that is a cumulative indication of caries experience. According to this study, the children aged 3–5 years had a mean dmft of 5.41 ± 4.81. This has highlighted the lack of knowledge regarding the association between various sugar intake patterns and dental caries (Cianetti et al. 2017). All of these results point to a reduced level of knowledge regarding oral health and highlight the necessity of well‐designed dental education initiatives. Early pediatric dental visits are advised by the American Academy of Pediatric Dentistry and a number of independent research. These visits should preferably occur within the age of 6 months of primary teeth eruption or before 1 year, as suggested by the American Dental Association (Liska et al. 2019). The results of current investigation were consistent with a prior study of Saudi Arabian school children, which found a mean dmft of 5.82 ± 4.48 (Mallineni et al. 2023). However, our findings were not as high as those of a prior Brazilian study that showed a mean dmft of 6.25 ± 4.20 among preschoolers (Vollú et al. 2018).

Mothers' level of knowledge and attitude are crucial for the prevention of oral diseases and the promotion of the oral health of their children. The majority of mothers in this survey displayed a low level of knowledge about their own as well as the dental health of their children. This result was in conflict with certain previous publications (Khaaviya et al. 2021; Shetty et al. 2016), but it was in line with some prior reports (Alsumait et al. 2019; Hamasha et al. 2019). An association was shown between the mothers' knowledge level and the oral health of their children. In addition, the low oral health knowledge of mothers was reflected in the oral health status of their children in terms of dmft scores, i.e., higher scores of dmft were shown by children of mothers having lesser knowledge as compared to those having higher knowledge. This suggested that mothers would highly benefit from efficient dental health education programs. Social media campaigns or educational initiatives might be a useful strategy for raising mothers' awareness (Moses and Arunachalam 2018).

No previous studies were conducted to compare mothers' attitudes and behavior toward their own oral health between public and private schools; however, the current results are in line with studies conducted in Rawalpindi city (Sajjad et al. 2016). They found the majority of teachers in private schools had significantly higher attitudes and knowledge regarding their own oral health as compared to the teachers in public schools. Studies conducted in Lagos State, Nigeria, found that students in private schools had better oral health practices compared to public schools (Soroye and Braimoh 2016). While another study conducted in Riyadh city found that there was no statistically significant difference among students in public schools versus students in private schools regarding self‐report of tooth brushing (Al Subait et al. 2016).

In this research, there were significant variations in the total mean knowledge scores of mothers with their education level. Mothers having high school education had an average knowledge score of 5.64 ± 1.83, whereas those who attended university had an average knowledge score of 6.93 ± 1.63. This suggested that mothers with only an elementary or secondary education may rely more on health education programs, whereas mothers with a university degree are more likely to look for information on their own from publicly accessible sources like the internet; however, it may include errors and be skewed toward a particular viewpoint. Thus, mothers' educational level may have a significant influence on the dental health of their children. Our findings concur with those of previous research studies in the literature (Bennadi et al. 2014; Mahmoud et al. 2017). The fact that mothers with greater levels of education frequently visited the dentist for dental checkups as opposed to a consultation further supports our findings. It is interesting to note that while scheduling dental checkups, highly educated mothers gave less thought to their child's willingness than less educated mothers. This could also be attributed to the autocratic and uncompromising nature of highly educated mothers in making decisions (Salama et al. 2020). On the other hand, a lower level of education is strongly associated with a diminished understanding of the importance of health and limited access to dental care. Furthermore, mothers with lower levels of education frequently spend their time in physical labor; as a result, they do not have sufficient time for dental examinations (Olak et al. 2018). The result that children of highly educated mothers had lower scores of dmft was further clarified by our study. This could be explained by the fact that parents with lower levels of education may not be as aware of the significance of preventative measures and oral hygiene as parents with greater levels of education (Nassar et al. 2022). This could also be due to the fact that these children had a greater tendency to brush their teeth more often, get more dental checkups, eat fewer sugary drinks, and had lower rates of dental caries (Chen et al. 2020). Our findings, however, conflicted with those of a previous study that discovered a significant relationship between higher maternal education and a higher frequency of caries (Ellakany et al. 2021). One possible explanation for this might be the working status of mothers, who may not have the time to develop good dietary practices and oral hygiene habits among their children (Fernando et al. 2019). A different explanation might be that mothers in Saudi Arabia who have a high socioeconomic status and are well‐educated are accustomed to eating fast food and sugar‐laden drinks (Alshehri 2016).

According to this study, children in private kindergartens had higher dental caries rates than children in public kindergartens. This might be explained by variations in the research population's oral hygiene habits, use of dental care services, and socioeconomic status. It is shown that students from private schools have superior oral hygiene habits and use of dental care. These outcomes agree with the results of previous reports (Soroye and Braimoh 2016). Furthermore, there was a statistically significant difference in the dmft scores between children in public and private kindergartens. The children's poor oral hygiene habits in public schools may possibly account for the disparity. The efficacy and consistency of oral self‐care routines, along with the frequent use of dental care services for the purpose of scaling and polishing, are strong determinants of oral hygiene status (Farsi et al. 2017). In this regard, socioeconomic status has a significant role in determining the usage of dental services; the higher the status, the more dental treatments are used. Children from high socioeconomic classes attend private preschools, and their mothers have sufficient education and knowledge and are thus more worried about their children's dental health and nutrition (Kamiab et al. 2021).

In this study, mothers with greater monthly incomes also scored lower on dmft and showed greater knowledge about the dental health of their children. According to earlier research, children whose parents had greater income had better oral health and a lower prevalence of dental caries (Hamasha et al. 2019; Zhang et al. 2020). These findings are consistent with our own. People from lower socioeconomic classes are less able to take care of themselves, get expert medical treatment, and live in a healthy environment because of social, financial, and material limitations. As a result, they are less resistant to oral and other illnesses. A low family income may have an impact on dietary choices and nutritional consumption (Ortiz et al. 2020). Our study results differ from those of research carried out in Timor‐Leste. The prevalence of caries was shown to be lower in children belonging to low‐income families because they showed reduced consumption of sugary snacks. Conversely, children from higher socioeconomic backgrounds had a higher caries prevalence because they consumed sweetened milk and calorie‐rich snacks more often. The possible explanation could be that in comparison to parents with higher education, parents with lesser education may get paid on a daily basis, and their children's frequent visits to the dentist could result in their losing their jobs or making it harder to schedule their work hours in addition to raising their children's knowledge of oral health (Sanguida et al. 2019).

The current investigation also demonstrated that younger mothers (≤ 30 years) had greater oral health knowledge scores than mothers in other age groups, as evidenced by their children's lower dmft scores. That might be because of the knowledge that has been disseminated through social media to aid and educate them since younger mothers tend to know more about dental health than older mothers do. The majority of younger mothers were aware of the significance of primary teeth and had the time to assist their children in brushing their teeth regularly than of older mothers. These findings corroborated the findings of the earlier study (Jain et al. 2014), but they disagreed with findings from studies carried out in Saudi Arabia and Pakistan, which found a significant correlation between older mothers and the impact of diet on their children's oral health (AlJameel et al. 2017; Iqbal et al. 2022). Mothers over 40 years old may have a greater number of children in their family, so they may learn about these issues from personal experience or even from records from prior dental appointments (Fernandes et al. 2017).

The strengths and limitations of this study should be considered when evaluating the findings. Compared to other research, the current study's response rate of 96% is very high (Fan et al. 2019; Naidu et al. 2016). Given that the sample comprised children from both public and private kindergartens in Jeddah, the results have the potential to be generalized to the whole population in Jeddah. The study participants were selected at random as well as a suitable sampling technique was employed to get a nearly representative sample. Furthermore, the questionnaire was pre‐validated, and a clinical assessment was conducted to describe the oral health status. This study's methodological features included a strong study design with good external as well as internal validity, sample distribution throughout different districts, a valid and reliable questionnaire, and suitable statistical analysis. One of the study's drawbacks was that the sample did not include children who did not attend kindergarten. Such types of mothers could think differently from mothers of children attending kindergartens. Another limitation was the reliance on self‐reported data during the completion of the questionnaires. Some participants may have misinterpreted the meaning of some questions. Participants may have offered more favorable responses rather than actual responses, to enhance their image, in spite of the anonymity of the questionnaire. In this research, a greater ratio of Saudi participants was present; if a non‐Saudi sample had also been included, the results might be differed. This study's cross‐sectional design is another drawback, and further research with a longitudinal design is advised in future research to produce more convincing data. Furthermore, the comparison between mothers' knowledge with any other guardian or fathers of the children was not made. Their viewpoints may warrant guidance for a potential behavioral intervention. Additionally, it is advised that further studies be done to compare children of different ages. The results of this study can assist in filling knowledge gaps and offer an update that can direct the development of caries prevention programs and community services for mothers and children by oral healthcare professionals.

Parents should plan the first dental visit before the age of 12 months, although it is often planned only in case of dental pain. There is a need for educating mothers regarding oral health status and reinforcing attitudes regarding oral health in Saudi Arabia. More health education programs and health promotions are needed among mothers. Our findings support the implementation of awareness campaigns/health education programs targeted at preschool children to encourage good oral health attitudes and behaviors. Additional studies are required to evaluate mothers' awareness regarding oral health in different cities of Saudi Arabia.

## 5 Conclusion

This study highlights the crucial role of maternal knowledge, attitudes, and behaviors in shaping children's oral health, with disparities observed between public and private kindergarten attendees. Lower maternal education and income levels were associated with higher dmft scores, emphasizing the need for targeted public health interventions. To address these disparities, integrating oral health education into prenatal and early childhood programs, implementing school‐based dental checkups, and providing accessible preventive dental care—particularly for lower‐income families—are essential. Preventive care and structured oral health programs in schools should be promoted to reach equitable and lifelong oral health habits among young children.

## Author Contributions

The study's conception and design were done by Prof. Najlaa M. Alamoudi, Prof. A. Eman Elashiry, Dr. Duha K. Qattan, and Dr. Nada O. Bamashmous. Data acquisition was done by Dr. Duha K. Qattan and analysis and/or interpretation of data by Dr. Osama M. Felemban. Drafting of the manuscript was done by Dr. Rana A. Alamoudi, while revising the manuscript was done by Prof. Najlaa M. Alamoudi, Dr. Ranan A. Alamoudi, Dr. Osama M. Felemban, Dr. Duha K. Qattan, and Dr. Nada O. Bamashmous. All authors approved the final draft of the manuscript.

## Ethics Statement

The research protocol was approved by the Research Ethics Committee at the Faculty of Dentistry, King Abdulaziz University, Saudi Arabia (Proposal Number: 008‐16).

## Conflicts of Interest

The authors declare no conflicts of interest.

## Data Availability

The data that support the findings of this study are available from the corresponding author upon reasonable request. The datasets generated during and/or analyzed during the current study are available from the corresponding author upon reasonable request.
